# Reduced microstructural white matter integrity is associated with the severity of physical symptoms in functional neurological disorder^☆^

**DOI:** 10.1016/j.nicl.2025.103791

**Published:** 2025-04-27

**Authors:** Nicolas Gninenko, Eliane Müller, Selma Aybek

**Affiliations:** a Faculty of Science and Medicine, Department of Neurology, University of Fribourg, Fribourg, Switzerland; b Department of Neurology, Psychosomatic Medicine Unit, Inselspital Bern University Hospital, University of Bern, Bern, Switzerland; c Graduate School of Cellular and Biomedical Sciences (GCB), University of Bern, Bern, Switzerland

**Keywords:** Diffusion-weighted imaging, Dissociative seizures, Functional movement disorder, Functional neurological disorder, Functional seizures, Microstructural integrity, White matter

## Abstract

**Background:**

Functional neurological disorder (FND) is linked to functional changes in brain networks without an underlying brain lesion. However, the dichotomy between functional and structural changes has been challenged by research suggesting that not only functional but also anatomical alterations in the gray and white matter may underlie a subset of symptoms. This study aimed to characterize white matter microstructural integrity and its association with patient-reported and clinician-rated physical symptoms’ severity in a large sample of FND patients.

**Methods:**

Diffusion-weighted imaging data were collected from 85 FND patients with mixed symptoms and 75 healthy controls (HCs), together with illness duration, clinician-rated (S-FMDRS & CGI), and patient-reported (SF-36) symptom severity. Microstructural integrity was computed based on probabilistic tractography using the Desikan-Killiany parcellation.

**Results:**

Compared to HCs, patients with FND presented widespread reduced microstructural integrity stemming from regions such as the right lateral orbitofrontal cortex, insula, putamen, and superior temporal regions. After adjusting for depression and anxiety, these differences were no longer significant. Within-group analysis revealed that reduced microstructural integrity, particularly in the left precuneus and left superior parietal cortex, was strongly correlated with both patient-reported and clinician-evaluated severity of physical symptoms in FND patients.

**Conclusion:**

Patients with FND present widespread reduced microstructural integrity in the brain, predominantly originating from temporoparietal, paralimbic and associated regions involved in emotion regulation and body awareness. These changes seem to be partly explained by comorbid mood disorders and the severity of physical symptoms, suggesting a plasticity phenomenon rather than trait biomarkers, which warrants further investigation in longitudinal study designs.

## Highlights

• Widespread reduced white matter integrity found in FND patients vs healthy controls.
• Physical symptom severity in FND patients linked to lower microstructural integrity.
• Between-group differences explained by comorbid anxiety and depression symptoms.

## 1 Introduction

Functional neurological disorder (FND) is a prevalent medical condition characterized by a wide range of clinical symptoms, including motor dysfunction (e.g., tremor, gait disturbance, weakness), sensory dysfunction (e.g., pain, numbness), cognitive dysfunction, functional/dissociative seizures and persistent postural-perceptual dizziness (PPPD) (Edwards et al., 2013, Espay et al., 2018, Hallett et al., 2022). The etiology of FND is multifaceted and involves a combination of psychosocial stress-related bodily dysregulation, possible (epi)-genetic predisposition, and abnormalities in brain information processing (Aybek et al., 2008, Ludwig et al., 2018, Apazoglou et al., 2017, Weber et al., 2023). Despite the growing body of neuroimaging research, the neuropathological understanding of FND remains limited (Perez et al., 2021, Bègue et al., 2019). The integration of multimodal neuroimaging approaches may be crucial in advancing our understanding of FND in an effort to identify robust biomarkers that can guide prognosis and inform biologically based therapeutic interventions (Perez et al., 2021).

As the macroscopic structure of the brain was thought to be largely preserved in FND patients, much of the research in FND has previously focused on functional neuroimaging. Functional magnetic resonance imaging (fMRI) studies, using both resting-state and task-based paradigms, have shown significant differences in brain activity and connectivity in FND patients compared to healthy controls (HCs) (Perez et al., 2021). These included increased connectivity in the amygdala and cingulo-insular regions with areas involved in motor control (Diez et al., 2019, Morris et al., 2017, Wegrzyk et al., 2017, Voon et al., 2010), hypoactivation and connectivity deficits in the temporoparietal junction (TPJ) (Baek et al., 2017, Maurer et al., 2016, Voon et al., 2010), as well as impairments in motor planning and intention (Voon et al., 2011, de Lange et al., 2010).

In recent years, research has also begun to uncover structural abnormalities in the brains of FND patients, challenging the traditional ‘software *versus* hardware’ analogy (Stone et al., 2016). Gray matter studies have identified reduced volumes in regions such as the cingulate-insular cortex (Labate et al., 2012, Perez et al., 2017, Perez et al., 2017), changes in amygdala volume (Weber et al., 2023, Maurer et al., 2018), and alterations in sensorimotor and striato-thalamic structures (Aybek et al., 2014, Sojka et al., 2022). More recently, white matter studies have also emerged in the field of FND. Diffusion-weighted imaging (DWI) represents a promising technique to investigate white matter abnormalities among structural alterations. DWI, particularly diffusion tensor imaging (DTI), quantifies the diffusion of water molecules in brain tissue, allowing researchers to study microstructural properties of white matter. Fractional anisotropy (FA), a scalar metric summarizing anisotropic diffusion, allows the differentiation of diffusion properties of various tissues. It can be used as an indicator of the microstructural integrity at specific locations in the brain, with reduced FA values indicating compromised white matter integrity. Using probabilistic tractography, white matter tracts can then be constructed and visualized (Jones et al., 2013). In turn, network-based analysis relying on graph theory allows for constructing connectivity matrices (i.e., connectomes) based on the obtained structural information, given that an appropriate brain parcellation is used.

Early DWI studies with small cohorts (n = 8, n = 16) of FND patients with functional seizures reported microstructural differences in the uncinate fasciculus (UF) and corona radiata compared to HCs, with a notable rightward asymmetry in the UF (Hernando et al., 2015, Lee et al., 2015). Age at disease onset was found to be inversely correlated with the degree of FA asymmetry in the UF. Another study with 17 patients with functional seizures reported altered structural connectivity in sensorimotor, attentional, subcortical, and default mode networks, suggesting widespread network dysfunction in FND (Ding et al., 2013). However, a different study found no group differences between 20 patients with functional seizures and 20 HCs (Jungilligens et al., 2021), but reported that mean FA values in the right fornix/stria and posterior corpus callosum were correlated with age at illness onset and seizure frequency.

In motor subtypes of FND (i.e., functional movement/motor disorder — FMD) (Park, 2024), a study with 44 patients with functional dystonia identified decreased FA in the splenium of the corpus callosum, brainstem, corticospinal tract, and other major white matter tracts, predominantly on the right side, using tract-based spatial statistics (Tomic et al., 2020). However, more recent studies using a transdiagnostic approach to mixed FND symptoms have produced disparate results. Sojka et al. (2021) reported decreased FA in the right mid-temporal white matter in a functional seizure subgroup (n = 21), but found no significant differences in the FMD subgroup (n = 17) compared with HCs (n = 38). Diez et al. (2021) used probabilistic tractography and graph-based methods in a mixed sample of 32 patients (n = 14 with functional seizures) and found reduced FA in several key limbic and associative fiber bundles, including the medial forebrain bundle, UF, cingulum bundle and corpus callosum. Smaller parts of these fiber bundles remained significant after correction for anxiety and depression (Diez et al., 2021). For example, reduced FA in the stria terminalis/fornix was found in patients with FND, but this reduction was mostly explained by comorbid anxiety and depression, as the difference did not remain significant after statistical correction. However, in within-group analyses, reduced FA in the stria terminalis/fornix correlated with larger physical disability and longer illness duration (Diez et al., 2021).

Although several DTI studies have reported white matter alterations in FND, the findings remain inconsistent — likely due to factors such as clinical heterogeneity, small sample sizes, methodological variability, and limited adjustment for comorbid mood disorders. Moreover, few studies have directly explored the relationship between broader patterns of white matter disruption and clinician-rated symptom severity. This relationship is of interest to understand whether the observed structural brain changes may represent trait biomarkers (independent of having a symptom) or state biomarkers (linked to the presence and severity of a symptom). To address these gaps, we examined microstructural differences using a global tractography approach in a large, clinically diverse cohort of FND patients and assessed their association with symptom severity, while carefully controlling for anxiety and depression. This method allows us to capture the full complexity of white matter organization, without being limited to preselected pathways — an important consideration given the distributed and variable nature of FND. Building on our previous finding of reduced hippocampal and amygdala volumes in this cohort (Weber et al., 2023), we hypothesized that FND patients would exhibit reduced white matter integrity in related association tracts, and that the degree of these alterations would correlate with both patient-reported and clinician-rated symptom severity.

## 2 Materials and methods

### 2.1 Participants

The available DWI data from a previously published cohort (Weber et al., 2023, Weber et al., 2024) of 85 FND patients diagnosed with motor (International Classification of Diseases, Tenth Revision [ICD-10] code F44.4) and sensory (F44.6) symptoms, functional seizures (F44.5), mixed symptom type (F44.7), and persistent postural-perceptual dizziness (PPPD, ICD-11 code AB32), as well as from 75 age- and sex-matched HCs, were further preprocessed. The FND diagnosis was established according to DSM-5 (American Psychiatric Association et al., 2013) and positive signs (Stone and Carson, 2015) by experienced board-certified neurologists from the University Hospital Inselspital (Bern, Switzerland). Subjects were excluded from the study if at least one of the following criteria was met: a major neurological comorbidity, a severe psychiatric condition (acute suicidality, active psychotic symptoms), history of alcohol or drug abuse, pregnancy or breast-feeding, contraindications to MRI, or insufficient proficiency in one of the Swiss national languages to understand the study protocol and provide informed consent. The study was approved by the local ethics committee of the Canton Bern (BASEC2017-00997) and conducted according to the principles of the Declaration of Helsinki. All enrolled subjects provided written informed consent. Additional details of the entire study protocol were previously documented (Weber et al., 2023).

### 2.2 Clinical assessments

Clinical assessments included the Clinical Global Impression (CGI) scale (Busner and Targum, 2007) (ranging from 0, no symptoms, to 7 among the most severely ill patients), the Simplified Functional Movement Disorders Rating Scale (S-FMDRS) (Nielsen et al., 2017), as well as the patient-reported Short-Form Health Survey (SF-36) (Ware and Sherbourne, 1992), from which four subscales (physical health, mental health, general health, and physical functioning) were used. The duration of symptoms was calculated in months from the date of symptoms onset to the date of inclusion in the study. The use of psychotropic (i.e., benzodiazepines, opioids, antidepressants, neuroleptics, and antiepileptics) or corticosteroid medication was recorded. Anxiety and depression were assessed using the State-Trait Anxiety Inventory (STAI, both trait and state) (Spielberger, 2010) and Beck Depression Inventory (BDI) (Beck et al., 1996), respectively.

### 2.3 Data acquisition

MRI data was acquired on a 3 T Siemens MAGNETOM Prisma scanner at the University Hospital Inselspital (Bern, Switzerland). Participants were allowed to watch an animal documentary and were instructed to remain calm and not to think of anything. Their heads were stabilized with foam pads to minimize head motion. Anatomical imaging was performed using a T1 magnetization prepared rapid gradient echo (3D-MPRAGE) sequence with a generalized autocalibrating partially parallel acquisition (GRAPPA, acceleration factor = 2) scheme, with repetition time (TR) = 2330 ms, anterior to posterior phase encoding (A≫P), echo time (TE) = 3.03 ms, inversion time (TI) = 1100 ms, 176 × 256 × 256 resolution (x × y × z), flip angle = 8°, isotropic voxel size (1.0 mm^3^), 176 volumes (∼5 min). DWI data was acquired in q-space using an interleaved echo planar spin echo (EPSE) diffusion-weighted sequence (acceleration factor = 2) with 30 directions, b_0_ = 0 s/mm^2^ (k-space average of three scans), b_max=30_ = 3000 s/mm^2^, TR = 3700 ms, A≫P, TE = 87 ms, 96 × 96 × 56 without gap, echo spacing = 0.58 ms, bandwidth = 2004 Hz/Px, and 2.2 mm^3^ isotropic voxel size (∼8 min).

### 2.4 Preprocessing of imaging data

**Fig. 1:**
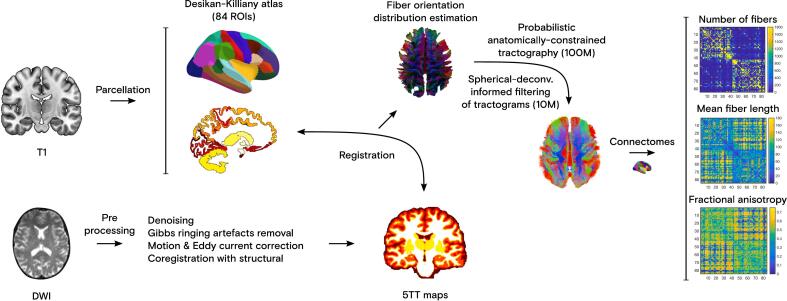
Diffusion-weighted imaging (DWI) and structural data (T1) preprocessing scheme. The anatomical images were segmented in FreeSurfer v7.4.1 to yield the Desikan-Killiany parcellation. DWI data was preprocessed in MRtrix3 v3.0.3 to yield the 5-tissue-type (5TT) maps, which are required for the fiber orientation distribution (FOD) estimation. Probabilistic tractography followed by spherical-deconvolution informed filtering (SIFT) of tractograms was performed to create structural connectomes (number of fibers, mean fiber length, and mean fractional anisotropy) for each subject.

Anatomical T1-weighted data was preprocessed using SPM12 (fil.ion.ucl.ac.uk/spm) in MATLAB R2021b (The MathWorks Inc., Natick, MA, USA) and segmented in FreeSurfer v7.4.1 (surfer.nmr.mgh.harvard.edu, ‘recon-all’ tool). Its gray matter was then parcellated into 84 brain regions (68 cortical and 16 subcortical) using the Desikan-Killiany atlas (Desikan et al., 2006). In addition, skull stripping was performed using FSL’s ‘bet2’ tool (with default options −f 0.5 −m), and in more challenging instances (e.g., when the −B option also failed), anatomical images were manually cropped in MATLAB (by zeroing-out obvious non-brain slices) prior to input to ‘bet2’. All anatomical data was visually inspected for quality control. Images were then reoriented to DWI space (using ‘flirt’, ‘transformconvert’, and ‘mrtransform’ tools) before applying 5-tissue-type segmentation using ‘5ttgen’. DWI data was further processed with MRtrix3 v3.0.3 (mrtrix.org) (Tournier et al., 2019), adapted from the standard Human Connectome Project (HCP) preprocessing pipeline. Processing steps included masking, Marchenko-Pastur principal component analysis (MP-PCA) denoising, Gibbs ringing artefacts removal, motion and Eddy current correction, intensity normalization, and coregistration to the anatomical volume using FSL v6 (fsl.fmrib.ox.ac.uk/fsl, ‘flirt’ tool) (Jenkinson et al., 2012). Susceptibility-induced distortion correction was not performed because reverse phase-encoded B0 images were not available. The data preprocessing pipeline is illustrated in Fig. 1. Detailed parameters and preprocessing code are available on GitHub (github.com/FND-ResearchGroup/DTI_BioGen_NG).

### 2.5 Tractography and microstructural integrity analyses

Probabilistic anatomically constrained tractography (ACT) was performed with 100 million streamlines using MRtrix3 (‘tckgen’ tool, with a maximum fiber length set to 250 mm, and a cutoff value at 0.06). Seeding was performed using the generated FOD map rather than a seed image. This approach was chosen for whole brain tractography to ensure that seeding density reflects the local white matter content. Specifically, we computed an FOD input (‘ss_wm.mif’) using ‘dwi2fod’ and applied instead the ‘-seed_dynamic ss_wm.mif’ option with ‘tckgen’. Outputs were then reduced to 10 % (10 million streamlines) through the spherical-deconvolution informed filtering (SIFT) (Smith et al., 2013) algorithm (‘tcksift’) and 84 × 84 structural connectomes were built (‘tck2connectome’) from the previously obtained Desikan-Killiany parcellation, namely number of fibers crossing each region, mean fibers length, and mean fractional anisotropy (FA) values (Fig. 1). To assess white matter microstructural integrity from each of the 84 regions, the weighted-degree (WD) graph metric was used as an integrative metric of local microstructural health, reflecting the cumulative strength of all connections originating from each region (Diez et al., 2021):

$${\mathrm{WD}}_{i}=\sum_{\begin{array}{c} j=1\\ j\neq i \end{array}}^{N}{\mathrm{FA}}_{i,j}$$

where *i* denotes a given region (node), *j* all other nodes (*j* ≠ *i*), and *N* = 84 regions from the used atlas. All scalar WD values per subject were therefore stored in vectors (of size 1 × 84), with each entry *i* representing the sum of all the FA values of the links starting in each node *i* and ending in each of the other nodes *j* = [1…*N*] (*j* ≠ *i*).

To assess differences in integrative microstructural integrity (WD) between HCs and patients with FND, general linear models (GLMs) were performed, accounting for age, sex, and additionally for psychotropic medication intake (see Table 1), depression (BDI) and trait anxiety (STAI-T) scores. Standard directional two-sample *t*-tests, owing to the distributions of data, were used to probe for any reduced or heightened microstructural integrity in FND patients against HCs (*p* < 0.05), with a Benjamini-Hochberg false discovery rate (FDR) correction at *q*_FDR_ = 0.05. To identify altered links, differences in whole-brain FA across all possible connections were similarly assessed using multiple regression, also accounting for age, sex, and additionally for the same depression and trait anxiety scores as above, and with the same statistical correction (*q*_FDR_ = 0.05). Only links pertaining to the previously identified brain regions showing group differences in WD were visualized in a connectogram. The same analysis was additionally performed for the number of streamlines, and the mean fiber length between both groups.

**Table 1:** Demographic, behavioral, and clinical characteristics of both groups. FMD: Functional Movement Disorder; PPPD: Persistent Postural-Perceptual Dizziness; CGI: Clinical Global Impression (scale); S-FMDRS: Simplified Functional Movement Disorders Rating Scale; SF-36: 36-Item Short Form Health Survey; BDI: Beck Depression Inventory; STAI-Y1/Y2: State-Trait (S/T) Anxiety Inventory. All metrics, unless otherwise specified, are reported as mean (standard deviation).

	FND participants (n = 85)	Healthy controls (n = 75)	*p*-value
Age, years	37.55 (14.26)	33.13 (10.97)	0.08
Sex (female/male)	63/22	55/20	0.9
Type of symptoms (Hallett et al., 2022)		*NA*	
FMD	44 sensorimotor 25 gait disorder 16 tremor 12 myoclonus 8 dystonia 5 speech disorder6		
Functional seizures	13		
PPPD	6		
ICD-10 classification	34 F44.4 5 F44.5 3 F44.6 37 F44.7 6 PPPD	*NA*	
Psychotropic medication	14 benzodiazepines 28 antidepressants 6 neuroleptics 9 antiepileptics 6 opioids	None taken	
Illness duration, years	4.89 (6.08)	*NA*	
S-FMDRS score	8.54 (9.65)	*NA*	
Illness severity, CGI score	2.69 (1.59)	*NA*	
SF-36 physical health	30.59 (35.64)	93 (23.09)	**< 0.0001**
SF-36 mental health	58.92 (23.17)	76.53 (13.69)	**< 0.0001**
SF-36 general health	47.59 (20.90)	79.33 (14.67)	**< 0.0001**
SF-36 physical functioning	64.24 (25.12)	97.33 (6.06)	**< 0.0001**
BDI score	14.33 (9.96)	4.41 (6.13)	**< 0.0001**
STAI-Y1 (S) score	37.04 (10.91)	31.76 (7.15)	**< 0.001**
STAI-Y2 (T) score	45.22 (12.92)	33.93 (7.13)	**< 0.0001**

### 2.6 Relationship between FA, WD, and clinical scores

To investigate potential associations between clinical variables of interest and brain regions or connections showing differences in integrative microstructural integrity (WD) or FA, we conducted a multiple correlation analysis between the selected clinical measures and the identified regions and links. Clinical variables included the four subscales of the SF-36 questionnaire (physical health, mental health, general health, and physical functioning), depression (BDI) scores, and trait (STAI-T) and state (STAI-S) anxiety scores. Different analyses were carried out, within each group (HCs and FND patients separately), and one combining data from both groups. The analysis for the FND group also included illness duration, S-FMDRS, and CGI scores. Analyses were first adjusted for age and sex and then further adjusted for depression (BDI) and trait anxiety (STAI-T) scores in subsequent models. For each analysis, associated *p*-values were computed, and FDR multiple comparisons correction was applied (*q*_FDR_ = 0.05). For integrative microstructural integrity (WD), *p*-values were sorted, and only significant correlations with clinical variables were retained, focusing on gray matter brain origins (among the 84 brain regions of the Desikan-Killiany atlas) previously identified as showing differences between HCs and FND patients.

## 3 Results

### 3.1 Demographic and clinical characteristics

Anatomical and DWI data from 85 patients with FND (63 female, 22 male, mean age 37.55 years ± 14.26 [SD], average illness duration 4.89 ± 6.08 years) and 75 HCs (55 female, 20 male, mean age 33.13 ± 10.97 years) were preprocessed and analyzed with the previously described graph theory approach. The most common symptom types were sensorimotor deficit, gait disorder and/or tremor. Out of the 85 FND patients, 37 had a mixed phenotype. Groups did not differ in age or sex distributions (*p* > 0.05), but FND patients presented with significantly higher depression (BDI, *p* < 0.0001) and anxiety (STAI-S, *p* < 0.001; STAI-T, *p* < 0.0001) scores at baseline, as well as with significantly lower general health-related quality of life scores on four subscales from the SF-36 questionnaire (Table 1).

### 3.2 Reduced microstructural integrity in FND

**Fig. 2:**
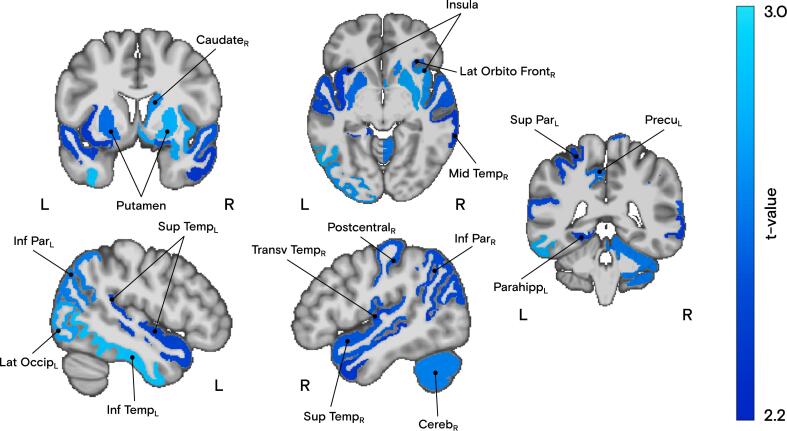
The nineteen brain gray matter regions depicted as nodes from the Desikan-Killiany atlas with reduced originating local microstructural integrity (weighted-degree [WD], as defined in the text) in patients with FND compared to HCs (*p* < 0.05), corrected for age and sex. The color bar represents the *t*-value of the two-sample *t*-test across all nodes (FDR-corrected for multiple comparisons, with *q*_FDR_ = 0.05).

Connectomes representing the number of fibers, mean fiber length, FA, and WD were constructed from the probabilistic tractograms (Fig. 1). WD analysis revealed significantly reduced local microstructural integrity stemming from 44 out of 84 brain regions in FND patients compared to HCs (*p* < 0.05 FDR-corrected with *q*_FDR_ = 0.05 for multiple comparisons using the Benjamini-Hochberg procedure). When adjusting for age and sex, 19 regions remained significant (Fig. 2), namely the right lateral orbitofrontal cortex, bilateral insula, right middle and transverse temporal cortices, left inferior and bilateral superior temporal cortices, left parahippocampus, right postcentral, bilateral inferior parietal, left lateral occipital, bilateral putamen, right caudate, left superior parietal, left precuneus, as well as the right cerebellar cortex. No regions were found with higher local microstructural integrity in FND patients than in HCs. WD differences between both groups became trends (at *p* < 0.05 FDR-corrected with *q*_FDR_ = 0.05) when BDI and STAI-T scores were also regressed out from the data before comparison, suggesting an intertwined effect of anxiety and depression in the FND population (lowest uncorrected *p* = 0.0357). When psychotropic medication intake was included in the model alongside age and sex, group differences also shifted to trend-level significance (under the same FDR-correction; lowest uncorrected *p* = 0.0025). This suggests a potential influence of medication, though the effect appears less pronounced and likely overlaps with that of mood comorbidities, given the frequent use of antidepressants and benzodiazepines in our FND cohort.

**Fig. 3:**
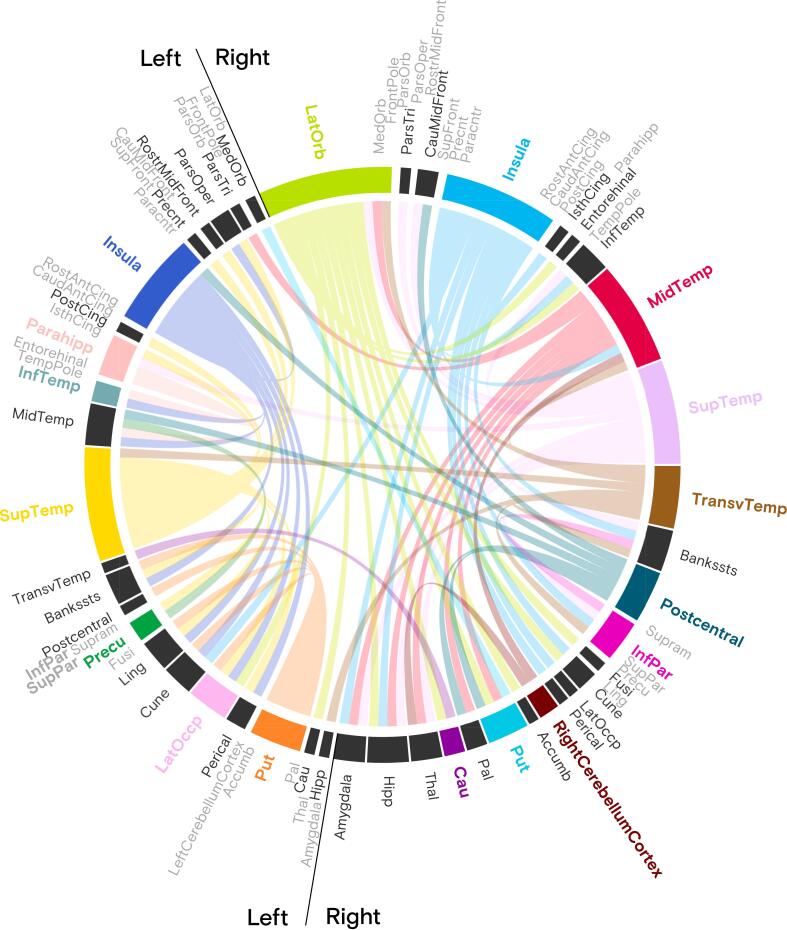
Connectogram representing reduced FA (links) between patients with FND compared to HCs (*p* < 0.05, FDR corrected at *q*_FDR_ = 0.05 for age and sex). Visualization is restricted to significantly altered links stemming from the previously identified 19 brain gray matter origins (in bold color) with decreased local microstructural integrity (WD) in FND. The left inferior and superior parietal cortices (in bold gray) show no significantly differing links. FA is more impacted in the right hemisphere, with a higher density of altered links. Regions labeled in light gray do not show any altered links with the previously identified 19 brain gray matter regions. Note that the coloring scheme is sequential and only for visualization purposes. Visualization created with the help of digraph chord chart (by Zhaoxu Liu on the MATLAB Central File Exchange).

FA analysis revealed a higher number of altered links in the right hemisphere (*p* < 0.05 FDR-corrected with *q*_FDR_ = 0.05, adjusted for age and sex), with the largest numbers of altered links involving the right lateral orbitofrontal cortex (n = 13/83), bilateral insula (n_left_ = 8, n_right_ = 11), and right middle temporal (n = 10) and bilateral superior temporal (n_left_ = 11, n_right_ = 10) cortices (Fig. 3). No altered links were found for the left inferior and superior parietal cortices (labeled in bold gray). Regions labeled in light gray (n = 35/84) were not found to differ in microstructural integrity between HCs and patients with FND, and those also did not display any altered connectivity.

Regarding the number of streamlines and mean fiber length, no significant differences were found when adjusting for age, sex, trait anxiety, and depression scores. When only adjusting for age and sex, 3 links with reduced number of streamlines in patients with FND compared to HCs survived FDR-correction at *q*_FDR_ = 0.05 (Table S1), but those did not overlap with findings from the FA analysis.

### 3.3 Reduced microstructural integrity is associated with physical symptoms’ severity in FND

**Fig. 4:**
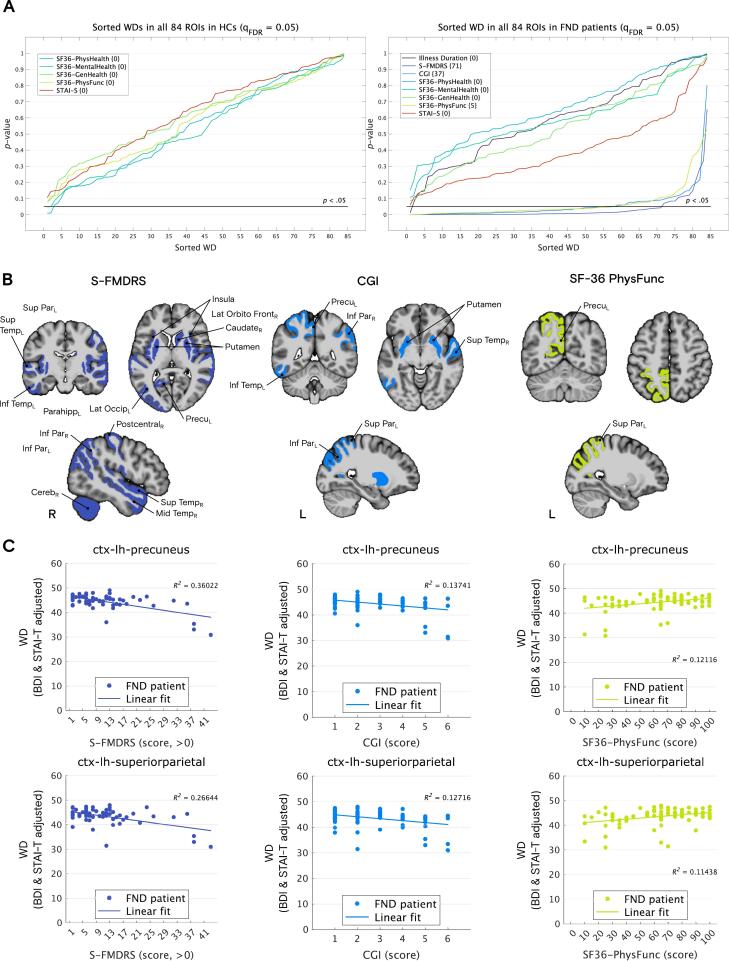
**A.** Sorted *p*-value correlation curves between clinical variables and local microstructural integrity values (WDs) in all 84 regions of interest (ROIs) of the Desikan-Killiany atlas for HCs (left) and FND patients (right). WDs are adjusted for age, sex, trait anxiety (STAI-T), and depression (BDI) scores. A line at *p* = 0.05 is shown. With FDR correction at *q*_FDR_ = 0.05, no associations survive for the HC group. For FND patients, WDs in many gray matter regions were significantly correlated (*p* < 0.05, FDR corrected at *q*_FDR_ = 0.05) with S-FMDRS scores (71 out of 84 ROIs), CGI scores (37 ROIs), and SF-36 physical functioning scores (5). **B.** Correlated gray matter regions were visualized on an MNI template brain, focusing on areas previously identified as showing significantly reduced local microstructural integrity between the two groups, resulting in 18 correlated regions for S-FMDRS, 8 for CGI, and 2 for SF-36 physical functioning. **C.** For all three clinical assessments, the left precuneus (top) and the left superior parietal cortex (bottom) were the gray matter origins with the strongest correlation between reduced local microstructural integrity and symptoms’ severity. Note that FND patients with no motor symptoms (i.e., an S-FMDRS score of 0; n = 21) were excluded from the visualization.

Given that a significant portion of the altered links identified earlier (Fig. 3; FA between patients with FND and HCs) were found to correlate with illness duration and clinical measures of physical symptom severity — specifically S-FMDRS, CGI, and SF-36 physical functioning (see Fig. S1 in the Supplementary Data) — we extended our analysis to include local microstructural integrity (WD) values as a complementary approach to explore whether these region-level metrics would also reflect relevant clinical associations. WD provides a higher-order, integrative measure of local connectivity by summarizing the total FA-weighted connections of each region, offering a broader view of regional microstructural integrity than individual tract-level FA values. A multiple correlation analysis across regions of interest (ROIs) between adjusted WDs and clinical variables revealed significant associations with S-FMDRS, CGI, and SF-36 physical functioning in several ROIs in the FND group. In the HC group, only SF-36 physical functioning showed a significant correlation in a single ROI. All results were FDR-corrected at *q*_FDR_ = 0.05. Interestingly, when data from both groups were combined, significant correlations were also found in several ROIs for depression (BDI scores) and SF-36 general and physical health scores (Fig. S2). However, when correcting for age, sex, depression (BDI), and trait anxiety (STAI-T) scores, no significant correlations were left in the HC group, but WD in several ROIs remained significantly associated with S-FMDRS (71 ROIs), CGI (37), and SF-36 physical functioning (5) scores in the FND group (Fig. 4**A**). When combining the corrected data from both groups, many associations with SF-36 physical functioning remained significant (Fig. S2). To restrict the results to previously identified regions showing significantly reduced local microstructural integrity in patients with FND compared to HCs (Fig. 2), filtered gray matter origins displaying significant correlations with S-FMDRS (18 out of 71), CGI (8 out of 37), and SF-36 physical functioning (2 out of 5) scores are plotted in Fig. 4**B**. For all three clinical measures, the most strongly correlated ROIs were the left precuneus and the left superior parietal cortex (Fig. 4**C**). Additional correlation plots for the remaining 16 ROIs for S-FMDRS and the remaining 6 ROIs for CGI are shown in Fig. S3 and Fig. S4, respectively. In summary, increased clinician-evaluated physical symptoms’ severity and decreased patient-reported physical functioning, as assessed by the S-FMDRS, CGI, and SF-36 physical functioning scales, were most strongly associated with reduced local microstructural integrity stemming from the left precuneus and left superior parietal cortex in FND patients.

## 4 Discussion

In this study, we identified widespread reduced local microstructural integrity across several brain regions in a cohort of 85 patients with FND compared to an age- and sex-matched HC group comprising 75 subjects. Gray matter origins with decreased local microstructural integrity included the bilateral insula, left parahippocampus, right postcentral, bilateral inferior parietal, left lateral occipital, right middle and transverse temporal, left inferior and bilateral superior temporal, right lateral orbitofrontal, and left superior parietal cortices, as well as bilateral putamen, right caudate, left precuneus, and right cerebellar cortex. Graph-theory analysis revealed that most links with reduced FA originated from the right middle temporal and bilateral superior temporal cortices, bilateral insula, and right lateral orbitofrontal cortex, with more affected links in the right hemisphere. However, these group differences became trends when adjusting for depression and trait anxiety scores, or for related medication intake (e.g., antidepressants and benzodiazepines), suggesting a strong intertwined effect of these comorbidities in FND. Consistent with our hypothesis, severities of patient-reported as well as clinician-evaluated physical symptoms were negatively correlated with local microstructural integrity, particularly in the left precuneus and left superior parietal cortex.

The bilateral superior temporal and inferior parietal regions (as well as the left superior parietal region), as defined in the used parcellation, partly overlap with the temporoparietal junction (TPJ) (Zito et al., 2020, Bühler et al., 2024), although the exact anatomical borders and subparts’ roles remain debated (Doricchi et al., 2022). While we did not find any local microstructural integrity differences in the supramarginal gyrus — likely due to its extended definition in this parcellation — the observation that TPJ is part of gray matter origins with the most altered FA with other brain regions aligns with existing literature on FND. Several studies have shown both structural and functional abnormalities in the TPJ. The TPJ is crucial for several functions, such as self-agency, multimodal integration, theory of mind, and spatial attention reorientation (Doricchi et al., 2022). Sojka et al. (2021) reported similar white matter changes in FND patients with functional seizures, showing reduced FA in mid-temporal white matter, specifically along the inferior longitudinal fasciculus and arcuate fasciculus, predominantly originating from the right TPJ and inferior temporal gyrus, although no such differences were found in the FMD subgroup. At the functional level, hypoactivity of the right TPJ and reduced functional connectivity with primary sensorimotor areas have also been demonstrated (Maurer et al., 2016, Voon et al., 2010, Zito et al., 2020), which may explain body awareness disturbances in patients with FND. These findings on the implication of TPJ as an origin of reduced white matter integrity with the likely involvement of major association tracts such as the arcuate fasciculus, inferior longitudinal fasciculus, and superior longitudinal fasciculus, may further contribute to the understanding of the neuroanatomical basis for the sense of agency deficits observed in FND patients.

The insula, an important hub for multisensory integration and processing of interoceptive information as well as self and emotional awareness (Craig, 2002), showed bilaterally reduced FA in patients with FND compared to HCs. This is consistent with previous neuroimaging findings linking structural and functional connectivity of the insula to symptom severity and emotional dysregulation in FND (Perez et al., 2017, Perez et al., 2017, Li et al., 2015, Espay et al., 2018). Anatomically, these alterations may reflect compromised integrity in white matter tracts such as the extreme capsule and UF, which connect the insula to the orbitofrontal cortex and anterior temporal regions, respectively (Schmahmann, 2009). Our findings support the view that aberrant structural connectivity in the insula may disrupt its functional integration, affecting the ability of patients with FND to accurately process bodily sensations, potentially leading to misinterpreted internal signals and distorted self-perception. Moreover, research has shown that FND patients exhibit increased stepwise functional connectivity from motor regions to bilateral insula (Diez et al., 2019), suggesting that disrupted connections between the insula and motor control regions may contribute to motor dysfunction and emotional disturbance. Given that our cohort predominantly includes FND patients with motor symptoms, this finding highlights the potential significance of motor-insular connectivity in the manifestation of their symptoms.

Reduced FA originating from the right lateral orbitofrontal cortex, a critical hub for decision making, risk assessment, emotion regulation, and social cognition (Rolls et al., 2020), have also been observed in patients with FND. This is in accordance with a recent DTI study by Diez et al. (2021) in FND patients with mixed symptoms. Authors found reduced FA in limbic tracts, including the UF, that connects the orbitofrontal cortex to temporal areas (Diez et al., 2021). However, studies with patients with functional seizures have shown different results. One study reported increased FA in the UF using tract-based spatial statistics (Lee et al., 2015), while Hernando et al. (2015) found no differences in mean FA in the UF between patients with functional seizures and HCs, but reported a significantly greater number of UF streamlines in the right hemisphere tract. Overall, these findings suggest altered white matter structural connectivity in emotion regulation pathways (involving the UF), originating from the lateral orbitofrontal cortex, although further research is needed to clarify the disparate findings among different FND subtypes.

In our cohort, patients with FND showed a tendency toward more white matter abnormalities in the right hemisphere. The right hemisphere is primarily involved in emotional processing, attention, and bodily awareness, with the latter frequently being impaired in FND, leading to dissociative symptoms and functional dissociation between bodily sensations and conscious perception (Devinsky, 2000). Previous studies in patients with functional seizures have also shown a higher frequency of structural lesions or physiologic dysfunctions in the right hemisphere (Devinsky et al., 2001), as well as reduced cortical thickness and disrupted white matter (Labate et al., 2012). These lateralized disruptions in corticolimbic pathways may contribute to the decoupling of emotional responses from sensory and perceptual processes, potentially exacerbating FND symptoms such as dissociation and bodily disconnection.

After adjusting for trait anxiety and depression scores, or for related medication intake (e.g., antidepressants and benzodiazepines), the observed local microstructural differences no longer remained statistically significant when applying strict FDR correction. This is however unsurprising, as FND patients often have comorbid psychiatric conditions such as anxiety and depression (Nicholson et al., 2020, Stone et al., 2010), which can complicate the interpretation of neuroimaging findings. The intertwined effect of these comorbidities was also shown in a previous DTI study by Diez et al. (2021), in which trait anxiety and depression affected probabilistic tractography differences in several fiber bundles, notably in the stria terminalis/fornix and UF. In this study, reduced FA from the lateral orbitofrontal cortex, insula, TPJ, and putamen were no longer significant when correcting for trait anxiety and depression scores. Since most of these regions are associated with emotional processing (i.e., the regulation and integration of emotional and somatic states), the observed structural differences could be linked to the impact of anxiety and depression on the brain. For example, abnormal activity and connectivity in the lateral orbitofrontal cortex have been associated with depression severity (Zhang et al., 2024, McCabe et al., 2008, Xie et al., 2021), while altered structural and functional connectivity of the insula have been linked to both anxiety and depression (Baur et al., 2013, Peng et al., 2018, Avery et al., 2014). These findings underscore the important role of emotional dysregulation in the pathophysiology of FND, as structural brain changes in areas responsible for emotional processing and regulation appear to reflect the common comorbid psychiatric conditions in patients with FND. This highlights the importance of including patient controls with other neurological and psychiatric disorders, besides HCs, to more accurately identify structural alterations specific to FND.

Reduced microstructural integrity in the left precuneus and superior parietal cortex correlated with both patient-reported (SF-36 physical functioning) and clinician-evaluated (S-FMDRS and CGI) symptom severity in FND, suggesting disruptions in key cognitive and sensory processes. The precuneus, a central hub of the default mode network (DMN), is involved in spatial awareness, episodic memory retrieval, the experience of agency, and emotion regulation (Cavanna and Trimble, 2006, Fransson and Marrelec, 2008, Lundstrom et al., 2005). White matter tracts supporting these processes likely include the cingulum bundle, connecting the precuneus to medial prefrontal and limbic structures (Wu et al., 2016). Previous studies with FMD patients have shown increased activation in the precuneus during emotion regulation tasks (Sojka et al., 2019) and a correlation between brain connectivity changes in the precuneus and functional weakness (Mueller et al., 2022). Children and adolescents with FND also showed hyperactivation of the DMN and sensorimotor networks (Kozlowska et al., 2017). Similarly, the superior parietal cortex integrates sensory information and directs attention, and its disruption can impair proprioception, body awareness, and sensorimotor coordination (Felician et al., 2004). In patients with functional seizures, disease duration was inversely correlated with cortical thickness of the superior parietal cortex and precuneus (Zelinski et al., 2022). Although we did not find a significant relationship with illness duration in our data, these findings confirm the role of the precuneus and superior parietal cortex in the altered brain function of patients with FND, potentially contributing to the alterations in sensory integration and cognitive control associated with this disorder.

An open question remains on how best to contextualize the FA differences identified in this study and their correlation with clinician-evaluated symptom severity — specifically, whether these findings reflect disease mechanisms, predisposing vulnerabilities, psychiatric comorbidities, compensatory processes, or a combination of these factors.

### 4.1 Study limitations and future directions

As noted earlier, psychiatric comorbidities, including trait anxiety and depression, are commonly associated with FND. Despite a carefully matched HC group, our study lacked a clinical comparison group, limiting our ability to determine whether the observed microstructural integrity changes were specific to FND. The results were also largely driven by FND patients with motor symptoms (∼78 % of patients), with only a small contribution from patients with functional seizures (13 patients) or with PPPD (6), which is insufficient for further subgroup analyses. Future studies involving a more diverse patient sample and a clinically relevant comparison group (e.g., psychiatric controls) may help to clarify the relationships between structural changes and specific clinical features of FND. Comparing our results to previous DTI studies in FND is also challenging due to the phenotypic variability of the patient population and subtle methodological differences (such as the unavailability of reverse phase-encoded B0 images for susceptibility distortion correction in our case). Additionally, alternative tractography schemes to ACT (e.g., global or ensemble tractography) may provide a more robust set of streamlines, although those may require more computational resources. Integrating white matter query language to map altered connections onto anatomically defined white matter tracts (Wassermann et al., 2016) may facilitate comparisons with prior findings, although this approach also has its own set of limitations. Moreover, the observed structural alterations are difficult to contextualize in the scope of predisposing vulnerabilities and compensatory processes in FND. Longitudinal interventional studies in FND patients should also include structural imaging when feasible, so potential prognostic biomarkers and neuronal mechanisms underlying treatment response can be identified.

## 5 Conclusion

We identified widespread reduced local microstructural integrity in our FND population compared to HCs, originating predominantly from temporoparietal, paralimbic and associated regions implicated in emotion regulation and body awareness. These regions are commonly associated with the pathology of FND and seem to be tightly linked to known comorbidities of FND, namely anxiety and depression. Reduced local microstructural integrity stemming from several brain areas, including the left precuneus and left superior parietal cortex, was inversely correlated with both patient-reported and clinician-evaluated physical symptoms’ severity. Our findings suggest that the pathophysiology of FND should be increasingly considered on anatomical grounds and that non-motor symptoms may deserve further investigation in larger structural imaging studies.

## CRediT authorship contribution statement

**Nicolas Gninenko:** Writing – review & editing, Writing – original draft, Visualization, Validation, Software, Resources, Project administration, Methodology, Investigation, Formal analysis, Data curation, Conceptualization. **Eliane Müller:** Writing – review & editing, Writing – original draft, Investigation. **Selma Aybek:** Writing – review & editing, Resources, Project administration, Funding acquisition, Conceptualization.

## Funding

This work was supported by the Swiss National Science Foundation (SNF grant PP00P3_210997 for SA).

## Declaration of competing interest

The authors declare that they have no known competing financial interests or personal relationships that could have appeared to influence the work reported in this paper.

## Data Availability

Data will be made available on request.
