# Supplementary material for: Reduced microstructural white matter integrity is associated with the severity of physical symptoms in functional neurological disorder
Source: Neuroimage Clin. 2025 Apr 27;46:103791. doi: 10.1016/j.nicl.2025.103791 (PMC12090312; doi:10.1016/j.nicl.2025.103791)
Supplement: Supplementary Data 1 [file mmc1.docx]

SUPPLEMENTARY DATA

Supplementary Data to:

Gninenko N, Müller E, Aybek S. Reduced microstructural white matter integrity is associated with the severity of physical symptoms in functional neurological disorder.

**Figure S1**. **A**. Sorted *p*-value correlation curves between clinical variables and FA values (links, restricted between all significantly altered gray matter origins (ROIs) of the Desikan-Killiany atlas between HCs and patients with FND — see Figure 2 in the main text) for HCs (left) and patients with FND (right). Top plots illustrate uncorrected data for age, gender, BDI, and STAT-T. In that case, several associations (e.g., 10 links with BDI in HCs) survive multiple comparison correction at *q*_FDR_ = 0.05. **B**. The same curves are plotted with FA values corrected for age, gender, BDI, and STAI-T. This shows that the effect of physical symptoms severity as evaluated by SF36-PhysFunc, CGI, and S-FMDRS, is widely sustained in the FND group (right), even after accounting for confounding effects of (trait-)anxiety and depression. To better identify the potential associations between gray matter origins showing reduced microstructural integrity and clinical variables, the weighted-degree (WD) analysis was performed at a coarser level (Figure 4 in the main text).

**Figure S2**. **A**. Sorted *p*-value correlation curves between clinical variables and microstructural integrity values (WDs) in all 84 regions of interest (ROIs) of the Desikan-Killiany atlas, for HCs’ and FND patients’ data grouped together. Left plot illustrates uncorrected data for age, gender, BDI, and STAT-T. **B**. The same curves are plotted with WD values corrected for age, gender, BDI, and STAI-T. When grouped together, only SF36-PhysFunc is significantly associated to microstructural integrity values stemming from 59 out of 84 ROIs across HCs and FND patients (FDR corrected at *q*_FDR_ = 0.05).

**Figure S3**. Individual subplots depict the remaining 16 gray matter origins with reduced microstructural integrity in FND patients compared to HCs, showing an association between WD values adjusted for age, gender, BDI, and STAI-T, and S-FMDRS scores of patients from the FND group. Note that FND patients with no motor symptoms (i.e., an S-FMDRS score of 0; n = 21) were omitted from the visualizations.

**Figure S4**. Individual subplots depict the remaining 6 gray matter origins with reduced microstructural integrity in FND patients compared to HCs, showing an association between WD values adjusted for age, gender, BDI, and STAI-T, and CGI scores of patients from the FND group.
